# Supplementary material for: Systematically optimized BCMA/CS1 bispecific CAR-T cells robustly control heterogeneous multiple myeloma
Source: Nat Commun. 2020 May 8;11:2283. doi: 10.1038/s41467-020-16160-5 (PMC7210316; doi:10.1038/s41467-020-16160-5)
Supplement: Supplementary file 3 — Reporting Summary [file 41467_2020_16160_MOESM3_ESM.pdf]

nature research

Corresponding author(s): Yvonne Y. Chen

Last updated by author(s): Apr 2, 2020

Reporting Summary

Nature Research wishes to improve the reproducibility of the work that we publish. This form provides structure for consistency and transparency in reporting. For further information on Nature Research policies, see Authors & Referees and the Editorial Policy Checklist.

Statistics

For all statistical analyses, confirm that the following items are present in the figure legend, table legend, main text, or Methods section.

n/a	Confirmed	
☐	☒	The exact sample size (*n*) for each experimental group/condition, given as a discrete number and unit of measurement
☐	☒	A statement on whether measurements were taken from distinct samples or whether the same sample was measured repeatedly
☐	☒	The statistical test(s) used AND whether they are one- or two-sided<br>*Only common tests should be described solely by name; describe more complex techniques in the Methods section.*
☐	☒	A description of all covariates tested
☐	☒	A description of any assumptions or corrections, such as tests of normality and adjustment for multiple comparisons
☐	☒	A full description of the statistical parameters including central tendency (e.g. means) or other basic estimates (e.g. regression coefficient) AND variation (e.g. standard deviation) or associated estimates of uncertainty (e.g. confidence intervals)
☐	☒	For null hypothesis testing, the test statistic (e.g. *F*, *t*, *r*) with confidence intervals, effect sizes, degrees of freedom and *P* value noted<br>*Give P values as exact values whenever suitable.*
☒	☐	For Bayesian analysis, information on the choice of priors and Markov chain Monte Carlo settings
☒	☐	For hierarchical and complex designs, identification of the appropriate level for tests and full reporting of outcomes
☒	☐	Estimates of effect sizes (e.g. Cohen's *d*, Pearson's *r*), indicating how they were calculated

Our web collection on statistics for biologists contains articles on many of the points above.

Software and code

Policy information about availability of computer code

Data collection	Flow cytometry data were collected using pre-installed software on the Macsquant VYB machine (Miltenyi, ver 2.11). Cell lysis data were collected using IncuCyte ZOOM2016B Software (Essen Bioscience). Bioluminescence imaging for animal studies was collected using Living Image Software version 4.4 (Perkin Elmer). Amplicon DNA sequencing data were collected using MiSeq Control Software version 2.6.2.1 (Illumina).
Data analysis	Flow cytometry data were analyzed using Flowjo (TreeStar). Lysis data were analyzed using InuCyte ZOOM Software (Essen Bioscience) and kill rates were determined using R 3.5.3 Software. FCAP Array v3.0.1 was used to analyze data collected with the BD Cytometric Bead Array kit. Bioluminescence images from animal studies were analyzed using Living Image Software version 4.4 (Perkin Elmer). Raw amplicon DNA sequencing data were analyzed using the DADA2 (ver 1.12) package on R 3.5.0 Software. Statistics were calculated using Microsoft Excel. Data plots were generated with either Microsoft Excel (Microsoft Office 365 version 2003) or GraphPad Prism 8.0.2 software.

For manuscripts utilizing custom algorithms or software that are central to the research but not yet described in published literature, software must be made available to editors/reviewers. We strongly encourage code deposition in a community repository (e.g. GitHub). See the Nature Research guidelines for submitting code & software for further information.

Data

Policy information about availability of data

All manuscripts must include a data availability statement. This statement should provide the following information, where applicable:

- Accession codes, unique identifiers, or web links for publicly available datasets
- A list of figures that have associated raw data
- A description of any restrictions on data availability

All data generated during this study are available from the corresponding author upon reasonable request. The source data underlying Figures 2, 3c-e, 4b-d, and 5, as well as Supplementary Figures 2a, 3c, 5, 6, 7, 9, 10b, 12, 15, 16a-c, 17c-g, and 19 are provided as a Source Data file.

Field-specific reporting

Please select the one below that is the best fit for your research. If you are not sure, read the appropriate sections before making your selection.

☒ Life sciences ☐ Behavioural & social sciences ☐ Ecological, evolutionary & environmental sciences

For a reference copy of the document with all sections, see nature.com/documents/nr-reporting-summary-flat.pdf

Life sciences study design

All studies must disclose on these points even when the disclosure is negative.

Sample size	A statistical method was not used to determine sample size. For in vitro studies, a minimum of triplicates was chosen to allow for calculation of statistics. For animal studies, between 3 and 6 mice were included for each treatment group. Three animals were used in pilot studies to enable basic statistical analysis. Six animals were used in full-scale studies based on our prior experience with the MM.1S tumor model indicating this sample size would likely enable detection of statistically significant differences in survival across different CAR designs based on log-rank analysis across test groups.
Data exclusions	No in vitro data was excluded. To ensure accurate analyses of samples collected from animal studies, tumor mass, tissue (e.g., brain and spleen), and peripheral blood samples with <100 EGFP+ tumor cells (for tumor-cell analysis) or <10 CD45+ cells (for T-cell analysis) were excluded. These exclusion criteria were pre-established based on the rationale that flow cytometry data with events fewer than the cut-offs indicated are unlikely to be reliable. The cut-off was set 10-fold lower for CD45+ cells compared to tumor cells because the CD45+ cell population was expected to be very limited across all samples.
Replication	All in vitro experiments were reliably reproduced, and the number of donors tested in independent experiments were indicated in the figure legends. For animal studies, each experimental treatment group included multiple animals. Each in vivo study shown was performed once, but control samples (e.g., animals treated with mock-transduced T cells) were repeated across the experiments shown to demonstrate reproducibility.
Randomization	For in vitro experiments, samples were allocated to identical cell-culture flasks/wells in a spatial pattern that facilitated organization. There is no reason to believe the spatial location of the sample influenced experimental results. In animal studies, mice were assigned randomly to experimental and control groups. Prior to T-cell injection, mice were distributed so that the overall tumor burden in terms of average radiance was consistent across different treatment groups.
Blinding	Studies were unblinded due to the need to make decisions on next-steps in the project based on emerging in vivo results, before long-term studies (132 days and 229 days in Figures 4 and 5, respectively) fully concluded. Care was taken to make sure that all samples were uniformly processed and analyzed to ensure consistency between control and test samples. For animal studies, mice in control and test groups were handled in a consistent manner to prevent study bias, and quantitative measures were used as much as possible in regards to determination of animal health (e.g., luciferase imaging, animal weight). Since the results reported are primarily quantitative (i.e., not subjective evaluation of behavioral changes, etc.), blinding was not necessary for the experiments.

Reporting for specific materials, systems and methods

We require information from authors about some types of materials, experimental systems and methods used in many studies. Here, indicate whether each material, system or method listed is relevant to your study. If you are not sure if a list item applies to your research, read the appropriate section before selecting a response.

Materials & experimental systems

n/a	Involved in the study
☐	☒ Antibodies
☐	☒ Eukaryotic cell lines
☒	☐ Palaeontology
☐	☒ Animals and other organisms
☒	☐ Human research participants
☒	☐ Clinical data

Methods

n/a	Involved in the study
☒	☐ ChIP-seq
☐	☒ Flow cytometry
☒	☐ MRI-based neuroimaging

Antibodies

Antibodies used	Antibodies that bind CS1-APC (BioLegend, cat no: 331809), BCMA-PE (BioLegend, cat no: 357503), anti-DYKDDDDK-APC (BioLegend, cat no: 637308), anti-HA-biotin (Miltenyi Biotec, cat no: 130-092-258), anti-HA-FITC (Miltenyi 130-120-722), Streptavidin-PE (Jackson ImmunoResearch, cat no: 016-110-084), PD-1-PE-Vio770 (Miltenyi Biotec, cat no: 130-117-698), LAG-3-APC (eBioscience, cat no: 17-2239-42), LAG-3-Brilliant Violet 421 (BioLegend, cat no: 369314), CD8-VioGreen (Miltenyi Biotec, cat no: 130-113-164), CD45-Pacific Blue (BioLegend, cat no: 304022), TCR α/β-biotin (BioLegend, cat no: 306704) were used in

the studies. In the animal experiment to evaluate anti–PD-1, Ultra-LEAF purified anti-human PD-1 antibody (BioLegend, cat no: 329959) was used.

Validation

Antibody identity was used as indicated by the manufacturers, which are identified in the Methods section. The manufacturers provided certificates of analysis; no additional validation was performed in house. For flow cytometry experiments, titration was performed for each antibody to determine the appropriate staining concentration. Positive and negative samples were stained at varying dilutions. In the case where negative samples were not available, corresponding isotype control antibodies were used. The staining concentration used had the largest dynamic range between positive and negative samples.

Eukaryotic cell lines

Policy information about cell lines

Cell line source(s)

Primary T cells were isolated from healthy donor blood from the UCLA Blood and Platelet Center. CD8+ cells were isolated using the RosetteSep Human CD8+ T Cell Enrichment Cocktail (StemCell Technologies) following manufacturer's protocols. Bulk T cells were isolated using RosetteSep Human T cell Enrichment Cocktail (StemCell Technologies). Peripheral mononuclear blood cells (PBMCs) were isolated using Ficoll density-gradient separation, and NM T cells were subsequently isolated from PBMCs using magnetism-activated cell sorting (Miltenyi) to first deplete CD25- and CD14-expressing cells and next enrich for CD62L+ cells. HEK293T cells and MM.1S cells were obtained from ATCC. Parental K562 cells were a gift from Michael C. Jensen (Seattle's Children Research Institute).

Authentication

Following isolation of primary T cells, T cells were stained for CD3, CD4, or CD8 expression by flow cytometry. Naive memory T cells were also stained for absence of CD25 and CD14 expression and enriched CD62L expression. K562 cells engineered to express BCMA or CS1 transgenes were stained with BCMA and CS1 antibodies. MM.1S KO cells were also stained with BCMA and CS1 antibodies to validate successful knockout. Expression of all markers was validated using flow cytometry. HEK293T cells were obtained from ATCC and not separately authenticated by our group.

Mycoplasma contamination

Cells were tested every month for mycoplasma contamination. Cells that tested positive were discarded.

Commonly misidentified lines
(See ICLAC register)

No commonly misidentified cell lines were used in this study.

Animals and other organisms

Policy information about studies involving animals; ARRIVE guidelines recommended for reporting animal research

Laboratory animals

Male and female NSG mice were used in animal studies. Six- to eight-week-old mice were bred in house by the UCLA Department of Radiation and Oncology.

Wild animals

Wild animals were not used.

Field-collected samples

Field-collected samples were not used.

Ethics oversight

All in vivo experiments were approved by the UCLA Animal Research Committee.

Note that full information on the approval of the study protocol must also be provided in the manuscript.

Flow Cytometry

Plots

Confirm that:

☒ The axis labels state the marker and fluorochrome used (e.g. CD4-FITC).

☒ The axis scales are clearly visible. Include numbers along axes only for bottom left plot of group (a 'group' is an analysis of identical markers).

☒ All plots are contour plots with outliers or pseudocolor plots.

☒ A numerical value for number of cells or percentage (with statistics) is provided.

Methodology

Sample preparation

Samples from in vitro studies included primary T cells as well as immortalized cell lines. Samples from animal studies were collected from mice, tumors were cut into fine pieces, filtered through a 100 um cell strainer (Corning) and washed with PBS prior to antibody staining. Prior to flow cytometry, samples were washed in PBS + 2% FBS and stained with antibodies.

Instrument

Flow cytometry data were collected on the MacsQuant VYB. Cell sorting was performed at the UCLA Flow Cytometry Core Facility using a FACSAria (II) sorter.

Software

Data were collected with the Miltenyi MACSQuant VYB and its native operative system and software. Data files were analyzed using FlowJo for Windows.

Cell population abundance	Cell lines that were sorted were sorted to near purity (95-100%). Purity was verified by flow cytometry assessing surface expression (or lack thereof in the case of antigen knockout) of target antigens.
Gating strategy	Initial FSC-Area/SSC-Area gates were drawn to remove debris and dead cells. A subsequent FSC-Area/FSC-Height gate was drawn to indicate singlet events. Multicolor samples were compensated with the help of single-color controls.The boundaries between "positive" and "negative" cell populations were defined by either an identically treated true-negative sample or the same sample stained with an isotype control antibody in the case where a true-negative was not available.

☒ Tick this box to confirm that a figure exemplifying the gating strategy is provided in the Supplementary Information.
