# Supplementary material for: Systematically optimized BCMA/CS1 bispecific CAR-T cells robustly control heterogeneous multiple myeloma
Source: Nat Commun. 2020 May 8;11:2283. doi: 10.1038/s41467-020-16160-5 (PMC7210316; doi:10.1038/s41467-020-16160-5)
Supplement: Supplementary file 4 — Description of Additional Supplementary Files [file 41467_2020_16160_MOESM4_ESM.pdf]

Description of Additional Supplementary Files

Supplementary Data 1:
MiSeq results of BCMA and CS1 amplicons from genomic DNA isolated from cell line and recovered tumor samples
